# Scientific research in primary health care: Lessons learned from a pragmatic multicenter implementation study in 101 general practices in the Netherlands

**DOI:** 10.1017/S1463423625000258

**Published:** 2025-04-02

**Authors:** Moniek Koopman, Jorn Reijnders, Bastiaan Kietselaer, Pim van der Harst, Rykel van Bruggen, Geert-Jan Dinant, Rozemarijn Vliegenthart, Robert Willemsen

**Affiliations:** 1 Department of Radiology, University of Groningen, University Medical Center Groningen, Groningen, the Netherlands; 2 Department of Cardiology, Zuyderland Medical Center, Sittard-Geleen, The Netherlands; 3 Department of Cardiology, Mayo Clinic, Rochester, MN, USA; 4 University Medical Center Utrecht, Utrecht, the Netherlands. Department of Cardiology, Division Heart and Lungs, University of Groningen, University Medical Center Groningen, Groningen, the Netherlands; 5 Multicenter General Practitioners Organisation ‘HuisartsenOrganisatie Oost-Gelderland’, Apeldoorn, the Netherlands; 6 Department of Family Medicine, Maastricht University, Maastricht, the Netherlands

**Keywords:** Implementation, computed tomography, coronary artery disease, coronary calcium score, general practioners, primary care

## Abstract

In this short report, the challenges and lessons learned from implementing scientific research in primary care are discussed. It highlights the complexities of conducting studies in primary care, where ‘Lasagna’s Law’ rules too often. Using the CONCRETE trial – a pragmatic multicenter implementation trial – as an example, eight key elements are identified as important factors for successfully conducting scientific research in primary care, such as optimizing digital processes and improving engagement.

## Introduction

Coronary artery disease (CAD) is one of the leading causes of death worldwide and chest discomfort or chest pain is an indicator for the presence of CAD (WHO, 2022, Khan *et al.*, 2020). The challenge for the primary care physician (PCP) – often the first consulted physician in cases of chest pain – is to assess the presence or absence of CAD (NHG, 2020). Distinguishing between life-threatening and non-life-threatening causes of chest pain is essential for the treatment of patients, but this is particularly challenging in patients with atypical chest pain or non-specific thoracic complaints (Rutten *et al.*, 2004, Hoorweg *et al.*, 2017). An accurate diagnostic and prognostic tool could help the PCP in determining the likelihood of CAD and guide patients’ management. At this moment PCPs in the Netherlands do not have access to advanced imaging tests for CAD, such as the computed tomography coronary calcium score (CT-CCS). The CT-CCS is a sensitive test for CAD and has a high diagnostic and prognostic value in symptomatic patients (Koopman *et al.*, 2022b). The introduction of CT-CCS as a diagnostic test in primary care is being investigated in the CONCRETE trial, and is nearing its conclusion (Koopman *et al.*, 2022a). In this short report, we discuss and share the lessons learned from the complex process of implementing scientific research in primary care.

### CONCRETE

The CONCRETE trial is a pragmatic multicenter implementation trial investigating the efficacy of availability of CT-CCS as diagnostic test in primary care in comparison to the standard of care (SOC). A full description of the study has been previously published (Koopman *et al.*, 2022a). Following a cluster-randomized design, participating practices were randomized into either the control or the intervention group. The control group followed the SOC in accordance with the Dutch primary care guideline (NHG, 2020), which recommends referring patients with (a)typical chest pain to the cardiologist and considering non-cardiac diagnoses in patients with non-specific thoracic complaints. In the intervention group, the PCP was instructed to request CT-CCS for patients with atypical chest pain and, in case of doubt, also for patients with non-specific thoracic complaints.

The primary outcome was cardiovascular risk management (CVRM) registration at practice level. Patients were included into CVRM if they have or are at elevated risk of cardiovascular disease, therefore receiving lifestyle and/or medical treatment to mitigate future cardiovascular events (NHG, 2019). Secondary outcome measures included patient-related measures such as CAD diagnosis and downstream testing. Downstream testing involved diagnostic tests requested in secondary care by the cardiologist. The original power calculation was based on the inclusion of 10 patients per practice per year (a seemingly reasonable assumption based on the fact that PCPs see patients presenting with atypical AP or non-specific complaints on a weekly basis) (Rutten *et al.*, 2004, Koopman *et al.*, 2022a).

The trial was well-received by PCPs as a diagnostic tool that could guide patient assessment and management is highly anticipated in primary care. The trial imposed a minimal burden on patients: after informed consent, patients were required to complete four questionnaires over 24-month, each taking about 10 min. Patients were informed about the trial by their PCP and received an information brochure created with patient input to enhance readability. Patients could contact researchers by phone or email with any additional questions about the trial. SOC patients were referred to the cardiologist and typically underwent one or more diagnostic tests during at least one visit to the hospital or 1.5-line care facility. CT-CCS patients underwent a CT scan at the radiology outpatient clinic, lasting less than 10 min and involving low radiation exposure (0.5 to 1.0 mSv) (RIVM, 2019; Messenger *et al.*, 2016). The procedure was conducted within five working days after their PCP consultation and results including clear recommendations per CT-CCS category, were reported to the PCP within five days thereafter.

### Measures taken to improve implementation

Before launching of the trial, the researchers identified key elements for implementation that enhanced inclusion among PCPs and patients, based on literature and on their experience. Measures to optimize inclusion rates focussed on minimizing inclusion efforts and reducing thresholds. By aiming on creating routine instead of complicated actions to include patients, the main principles of the normalization process theory (NPT) were followed (Murray *et al.*, 2010, May *et al.*, 2009). NPT explores how social processes influence the embedding of innovations in practice through four mechanisms - coherence, cognitive participation, collective action, and reflexive monitoring. NPT emphasizes the need for continuous investment in order to let the intervention fit seamlessly into daily practice (Supplemental Table 1) (May and Finch, 2009, Murray *et al.*, 2010). As a consequence, multiple steps were taken prior to and during the trial to maximize the implementation of CONCRETE in day-to-day PCP practice and to optimize patient inclusion (Table 1). In this section, we describe eight key elements that emerged as well as the actions taken to meet the demands arising from those key elements:

**Table 1.** Actions to optimize inclusion of primary care physicians and patients

*Communications with primary care physician and patients*	
Newsletter	PCPs were kept informed of the latest trial information, such as the inclusion rate, through important communication channels, including newsletters from local collaborating multicenter organizations and updates from the researchers, on a quarterly basis.
Instructions and education	PCPs, their assistants and staff were informed about the trial and received education during regular local PCP meetings, as well as on mandatory education days for both PCPs and assistants.
Face to face meetings	The trial researchers visited the practices multiple times during the trial. During these meetings, all relevant parties at the PCP office were informed about the trial, and any questions could be answered directly.
Website	This was created to provide general information about the trial for PCPs, patients, and other interested parties.
Primary care physician and all employees of the practice	
Local delicacy	A local delicacy was provided to the PCP practice as a thank you to all employees for achieving the minimum inclusion target of 10 patients per year.
Desk accessory	A reusable tissue box featuring the CONCRETE logo was given to all participating PCPs as a visible reminder of their participation in the trial and to encourage the continued inclusion of patients.
Reimbursements	A small financial compensation was provided for the additional time investment by PCPs in the SOC group participating in the trial.
*Patients*	
Poster or message on a screen in the waiting room	To motivate patients waiting in the waiting room to discuss their interest in participating in the trial with their PCP during their consultation.
Information letter in the waiting room	A letter was given by the assistant to patients consulting the PCP for chest pain complaints. This allowed patients to read information about the trial while in the waiting room. If interested, they could discuss potential participation with their PCP during the consultation.
The Dutch Heart Foundation	The Dutch Heart Foundation offers an “I want to participate in a study” option on their website. Interested patients could apply through this platform. These patients received information about the trial from the trial researchers and were advised to discuss their willingness to participate with their PCP.

*Note*: PCP = primary care physician.

1. A research *subject* that resonates with PCPs (Bakkenist et al., 2003, Bateman, 2002).
  PCPs are interested in subjects that could reduce their workload and could offer better diagnostic tests than is currently the case, enhancing both ruling out and early detection of disease. CONCRETE met the generally expressed needs as pronounced by PCPs for a reliable, easily explainable test to assess CAD. PCPs actively reached out to join the trial, shown by the participation of 250 PCPs across 101 practices.
2. A *research protocol* that fits within the day-to-day practice of the PCP.
  From the outset, PCPs and patients were actively involved in designing a research protocol with visuals aids that minimally impacted their daily practice. Primary care experts from various regions in the Netherlands joined the trial’s steering committee and collaborated with researchers to develop and refine the protocol based on real-world practice. Patients contributed by advising on trial communication, language, and questionnaires.
3. Testing the *research* protocol in a pilot to optimize its success, before implementing it in all PCP practices.
  Before the research protocol was broadly implemented, it was tested in four practices. Valuable feedback from PCPs, nurse practitioners, and assistants was used to optimize the study protocol.
4. Instructions and education to all those involved in the study to optimize uptake of the research in daily practice.
  Before and during the trial, we repeatedly provided instructions and education on relevant trial elements (e.g., diagnostic tests) to participating PCPs, nurse practitioners, and assistants. This helped to integrate the research into daily practice.
5. Optimizing digital support.
  An IT specialist and a PCP developed a referral file adapted to the trial within the Dutch platform ‘ZorgDomein.’ This platform, used by PCPs for referrals and diagnostic test requests, was designed to facilitate the inclusion process. The ‘referral file’ made it easier for PCPs to refer patients for diagnostic testing and simplified the associated inclusion of patients by minimizing extra inclusion steps.
6. Additional support to recognize eligible patients.
  During the trial, potential SOC patients were sent to the cardiologist without being included in the study by participating PCPs, likely due to oversight as no specific trial actions were required for the SOC group. Nurses in outpatient hospital clinics and in the participating 1.5-line care clinic were instructed to identify and facilitate participation by informing PCPs about these eligible patients.
7. *Accessibility* of *researchers* for participating PCPs, nurse practitioners, and assistants.
  Throughout the trial, researchers maintained regular contact by visiting offices, and were available for questions via email and phone, and provided support including digital research materials.
8. A swift reaction in case of unexpected hurdles.
  In the trial’s first year, we encountered an unexpected SOC change due to a revision of the Dutch guideline for Stable Angina Pectoris (AP), recommending direct referrals to the cardiologist instead of exercise testing in cases of atypical AP (NHG, 2020; Rutten *et al.*, 2004). The SOC protocol was adjusted accordingly. The COVID-19 pandemic also caused delays, pausing the trial for a few months after consultation with PCPs and all stakeholders. Afterwards, the trial was restarted against a background of increasing workload as was generally reported by Dutch PCPs after the pandemic. To address this, researchers offered to take over the complete inclusion procedure. About a quarter of PCPs adopted this new approach.

### Contemplation for future scientific projects in primary care

**Figure 1.**
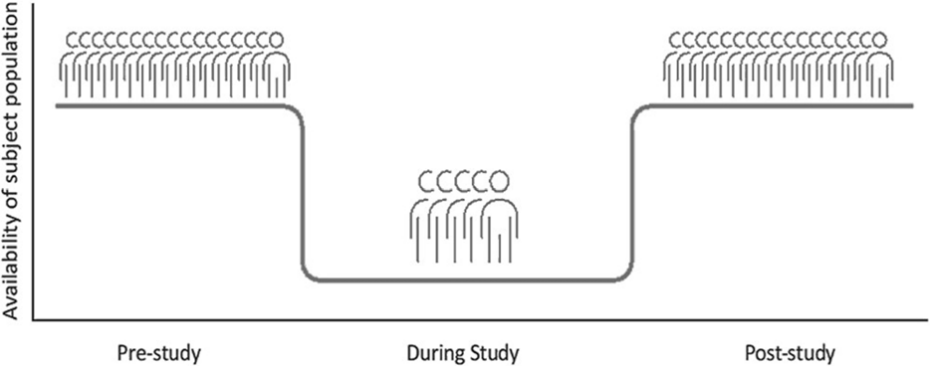
Visualization of ‘Lasagna’s Law’ in scientific research (Bogin, 2022).

Even after all these steps, the inclusion rate is lower than expected. Now, near the ending of the study, 549 patients were included in the intervention group and 131 in the control group. In literature, the phenomenon of inclusion rates dropping behind is often referred to as ‘Lasagna’s law’ (Figure 1) (Bogin, 2022). Indeed, literature describes that patient inclusion is often 10%-33% lower than calculated and every fifth trial is either stopped due to low inclusion or completed with less than 85% of the intended inclusion number (Carlisle *et al.*, 2015, Feinstein, 2001). This also applies to primary care, especially in studies that depend on incidental cases where the PCP is forced to perform inclusion actions during the consultation with the patient (van der Wouden *et al.*, 2007; Bogin, 2022). Other factors that contribute are: 1) incorrect estimates of the incidence of complaints and illness, 2) insufficient explanation or promotion of the study, 3) patient factors, and 4) inclusion and exclusion criteria or outcome measures that do not correspond sufficiently with day-to-day practice (Bogin, 2022). All these points have been addressed while setting up and running the CONCRETE trial.

Several studies have developed practical checklists to help researchers optimize inclusion rates. However, these have not been tested on relevant outcome measures in practice (Bateman, 2002; van der Wouden *et al.*, 2007; Huibers *et al.*, 2002). Although we will be able to present useful results in 2024/2025, numerous factors inhibiting seamless embedding in daily practice of study operations (as promoted in NPT, May *et al.*, 2009; Murray *et al.*, 2010) were encountered during CONCRETE: 1) disappointment among some PCPs due to randomization to the SOC group, reducing their engagement and protocol adherence; 2) time-consuming patient inclusion; and 3) a diminishing awareness of the study during its running time. These inhibiting factors, along with a pandemic, likely contributed to low inclusion rates (Supplemental Table 1). Yet, factors enhancing patient inclusion were also seen: 1) enthusiasm among participating caregivers to help developing a potentially cost-effective diagnostic test for CAD, and 2) a minimal time burden on PCPs and staff for patient inclusion.

Besides all the aforementioned barriers that apply to most studies, our CONCRETE trial faced unforeseen challenges, including a temporary halt during the COVID-19 pandemic and a delayed restart due to overload of PCPs post-COVID. Altogether, both routine issues and unexpected problems contribute to Lasagna’s phenomenon. An enhancement within the PCPs’ digital referral system (‘Zorgdomein’)—such as a reminder for referrals of patients with chest pain—might have improved our inclusion rates. Although considered, the cost to create this reminder were beyond the scope of this trial. For future research in the Netherlands, the General Practice Research Council is working on improving infrastructure to facilitate uniform digital systems for data follow-up and collection, easing pressures on PCPs and staff, and advancing primary care research (van Maanen *et al.*, 2023).

### Conclusion

Based on the multicentre CONCRETE trial, this short report illustrates that implementation of scientific research in primary care is as important as it is challenging. In a well-conducted study, researchers should – next to creating enthusiasm for the study – apply a multitude of actions to optimize inclusion rate. Moreover, due to the increased workload among PCPs, establishing the inclusion goals is partly beyond the investigators’ control. As a consequence, the process of inclusion is labour and cost-intensive, ‘Lasagna’s Law’ rules with a heavy hand and the performance of scientific research in primary care is under pressure.

## Supporting information

Koopman et al. supplementary material Koopman et al. supplementary material

## Supplementary material

The supplementary material for this article can be found at https://doi.org/10.1017/S1463423625000258
