# Supplementary material for: Scientific research in primary health care: Lessons learned from a pragmatic multicenter implementation study in 101 general practices in the Netherlands
Source: Prim Health Care Res Dev. 2025 Apr 2;26:e35. doi: 10.1017/S1463423625000258 (PMC12037340; doi:10.1017/S1463423625000258)
Supplement: Koopman et al. supplementary material [file S1463423625000258sup001.docx]

**Supplemental material**

| **Supplemental Table 1:** Evaluation of the implementation of the CONCRETE trial based on the framework of the Normalisation Process Theory (May and Finch, 2009, Murray et al., 2010) | | |
| --- | --- | --- |
| **Coherence (What is the work?)** | |  |
| Factors that promote or inhibit the mobilization of a practice/intervention; Beliefs and behaviours that define and organize objects; How is a practice/intervention conceptualized by participants? How does it hold together in action? | *Promoting factors:* 1) the opportunity to work with a new diagnostic test (CT-CCS) that has been proven effective in secondary care for detecting early stages of CAD; 2) the change to participate in scientific research that has the potential to change the SOC within primary care; 3) the opportunity to use a diagnostic test that could detect CAD in its early stages and possibly limit major cardiovascular events through early initiation of cardiovascular risk management; 4) an intervention that is less expensive than the SOC; and 5) participating in the trial or requesting the intervention will not extensively increase the time spent by PCPs or staffs on day-to-day activities.  *Inhibiting factors:* 1) not all participants (PCPs or PCP offices) will gain access to the new diagnostic test; they will continue to perform the SOC; and 2) participating in the trial will slightly increase the day-to-day time due to the need to explain the trial to patients, obtain their IC, send the information to the researchers, and request the diagnostic test (in case of the CT-CCS). |  |
| **Cognitive participation (who does the work?)** | |  |
| Factors that promote or inhibit participation in a practice; Beliefs and behaviours that define and organize actors; How do participants come to engage with a practice? How do they decide on engagements and the purpose that is serves? | *Promoting factors:* 1) performing both the CT-CCS intervention of SOC will not significantly increase the time spend by PCPs or staff to include patients, which involves obtaining the signed ICF and sending it to the researchers; 2) the explanation of the referral to a diagnostic test or cardiologist (SOC) is part of a normal PCP consultation, therefore, does not differ from the day-to-day activities.  *Inhibiting factors:* 1) PCPs and staff must take additional actions if patients want to participate in the trial; 2) staff needs to be informed about the trial by the PCPs; 3) staff must be informed of any additional actions required, such as obtaining the signed ICF and sending it to the researcher; and 4) staff must handle disappointment if the practice is not enrolled in the intervention group. |  |
| **Collective action (How does the work get done?** | |  |
| Factors that promote or inhibit enacting a practice; Beliefs and behaviours that define and organize work; How do participants enact a practice? How are their activities structured and constrained? | *Promoting factors: 1)* the trial did not actively disrupt the working mechanism of PCP offices, as it aligned with the existing structures and standard procedures for patients with atypical AP or non-specific complaints. For PCPs in the SOC group, no additional actions were needed concerning the referral procedure.  *Inhibiting factors: 1)* PCPs in the SOC group needed to invest some time to inform patients of the trial, obtain their signed ICF, and have a staff member send the ICF to the researchers; 2) PCPs in the SOC group and their staff needed to be remined, and remined themselves, about including patients in the trial; and 3) PCPs in the CT-CCS group needed to fill out (in the beginning) a referral form to request the CT-CCS. |  |
| **Reflexive monitoring (How is the work understood?)** | |  |
| Factors that promote or inhibit the appraisal of a practice; Beliefs and behaviours that define and organize understanding; How do participants appraise a practice; What are its effects of appraisal? How are they mediated? | Promoting factors: 1) PCPs in the trial had the opportunity to contribute to evidence that could change the future SOC for patients with atypical AP or non-specific complaints in primary care; 2) the trial provided a chance to gather evidence for a new intervention that could reduce healthcare-related costs; and 3) it allowed for the collection of evidence for the a new intervention that could detect CAD in earlies stages or provide reassurance to patients. Inhibiting factors: 1) PCPs in the CT-CCS groups needed to learn how to interpret the calcium scores and understand the related policies. |  |
| Note: CT-CCS= computed tomography coronary calcium scoring. SOC= standard of care. GP= general practitioner(s). ICF: informed consent form. | | |

MAY, C. & FINCH, T. 2009. Implementing, Embedding, and Integrating Practices: An Outline of Normalization Process Theory. *Sociology,* 43**,** 535-554.

MURRAY, E., TREWEEK, S., POPE, C., MACFARLANE, A., BALLINI, L., DOWRICK, C., FINCH, T., KENNEDY, A., MAIR, F., O'DONNELL, C., NIO ONG, B., RAPLEY, T., ROGERS, A. & MAY, C. 2010. Normalisation process theory: a framework for developing, evaluating and implementing complex interventions *BMC Med,* 8.
